# Catalytic nanomotors for environmental monitoring and water remediation

**DOI:** 10.1039/c4nr01321b

**Published:** 2014-04-22

**Authors:** Lluís Soler, Samuel Sánchez

**Affiliations:** a Max Planck Institute for Intelligent Systems , Heisenbergstr. 3 , 70569 Stuttgart , Germany . Email: sanchez@is.mpg.de

## Abstract

Self-propelled nanomotors hold considerable promise for developing innovative environmental applications. This review highlights the recent progress in the use of self-propelled nanomotors for water remediation and environmental monitoring applications, as well as the effect of the environmental conditions on the dynamics of nanomotors. Artificial nanomotors can sense different analytes—and therefore pollutants, or “chemical threats”—can be used for testing the quality of water, selective removal of oil, and alteration of their speeds, depending on the presence of some substances in the solution in which they swim. Newly introduced micromotors with double functionality to mix liquids at the microscale and enhance chemical reactions for the degradation of organic pollutants greatly broadens the range of applications to that of environmental. These “self-powered remediation systems” could be seen as a new generation of “smart devices” for cleaning water in small pipes or cavities difficult to reach with traditional methods. With constant improvement and considering the key challenges, we expect that artificial nanomachines could play an important role in environmental applications in the near future.

## Abstract 2

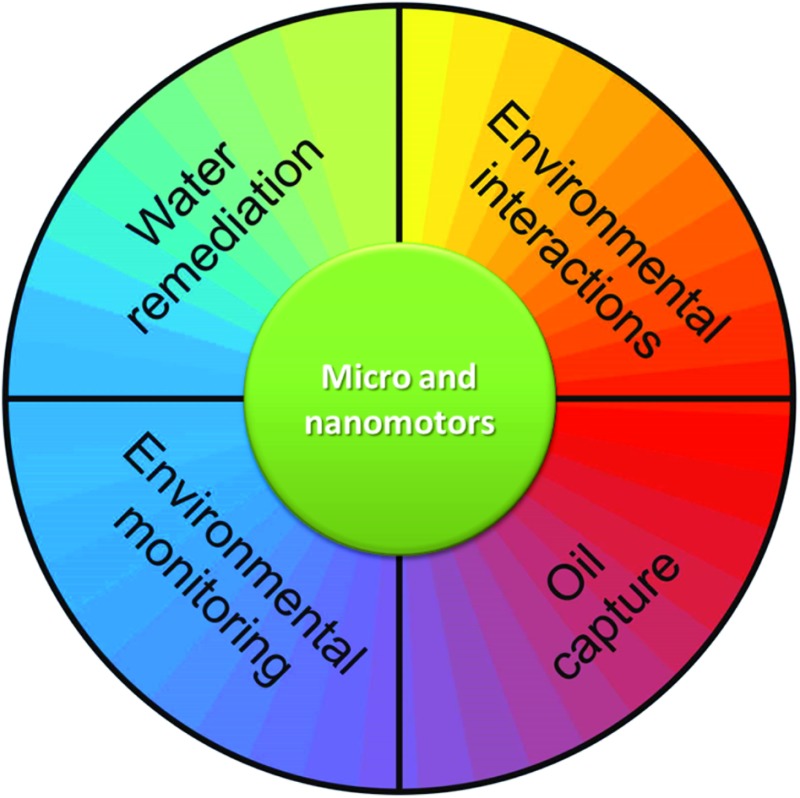
Self-propelled nanomotors hold considerable promise for developing innovative environmental applications.

## 1. Introduction

Pollution of water by contaminants and chemical threats is a prevalent topic in scientific, economic, political and, consequently, in the public media. The problems related to clean and safe water affect millions of people around the world as the number of contaminants originating from human activity is increasing over the last few years. Some examples are heavy metals; industrial products or chemicals such as solvents, additives or lubricants; personal consumer products such as detergents or pharmaceuticals; or pesticides.^1^ These contaminants cause problems that can range from contamination of drinking water to endocrine effects and bacterial resistance, which lead to human health problems.

Researchers and engineers are devoting considerable effort to produce more efficient technological solutions for cleaning environmental pollutants. Among these efforts are sustainable, lower-cost and energy-efficient methods for large-spectrum detoxification of chemicals, extended effectiveness, and the minimization of additional contaminants or chemicals that would endanger human health through the treatment itself. Ideally, these alternative methods should reach remote locations where standard detoxification methods are not capable to do so.^2^

The search for natural/green engineering methods for reducing energy and chemical usage, together with clean by-products, is sought after. For example, some ideal candidates for the next generation of water treatment are those that are able to remove pathogens and chemical threats, actively transport molecules, move ions against concentration gradients, separate compounds in complex media or deactivate chemical agents. Nanoparticles have been recently proposed as an alternative to improving or substituting standard filtration methods.^3^ Unfortunately, some limitations still exist like the recovery of particles from the solution after water treatment. This challenge motivated the use and development of "smart" materials, e.g. magnetically responsive nanoparticles, such as the recently reported nanoscavengers.^4^ Those magnetic-core nanoparticles contain antiferromagnetic core layers enabling rapid collection (less than 5 min) with a permanent magnet and enable the removal of contaminants from water. However, the transport of ions and non-active nature of these nanoparticles may be a downside because, for highly active remediation, external energy would still be required. In addition, those nanoparticles cannot transport ions and pollutants from one place to another.

Catalytically powered micro- and nanomotors have attracted a lot of attention over the last few years in multidisciplinary fields of chemistry and physics.^5^ Since the pioneering works a decade ago, synthetic nanomotors demonstrated the ability to efficiently convert chemical energy into motion like nature uses biochemistry to power biological motors.^6,7^ Fundamental research is being conducted in this field and a number of interesting applications are opening up in several different fields, such as the biomedical field^8^ and more recently the environmental field.^9,10^ Several approaches have been proposed to efficiently propel and accurately control micro- and nanomotors by different mechanisms.^11–18^ Self-propulsion of catalytic micromotors has been mainly demonstrated in the presence of hydrogen peroxide (H_2_O_2_) fuel, which decomposes into water (H_2_O) and oxygen (O_2_).^14,19–26^ Nevertheless, there are also a diversity of mechanisms to power micro and nanomotors, for instance photoinduced motion,^27–29^ electromagnetic fields,^30–32^ local electrical fields,^33^ thermal gradients,^34,35^ the Marangoni effect,^36^ ultrasound^37,38^ or biohybrid motion.^39,40^

**Fig. 1:**
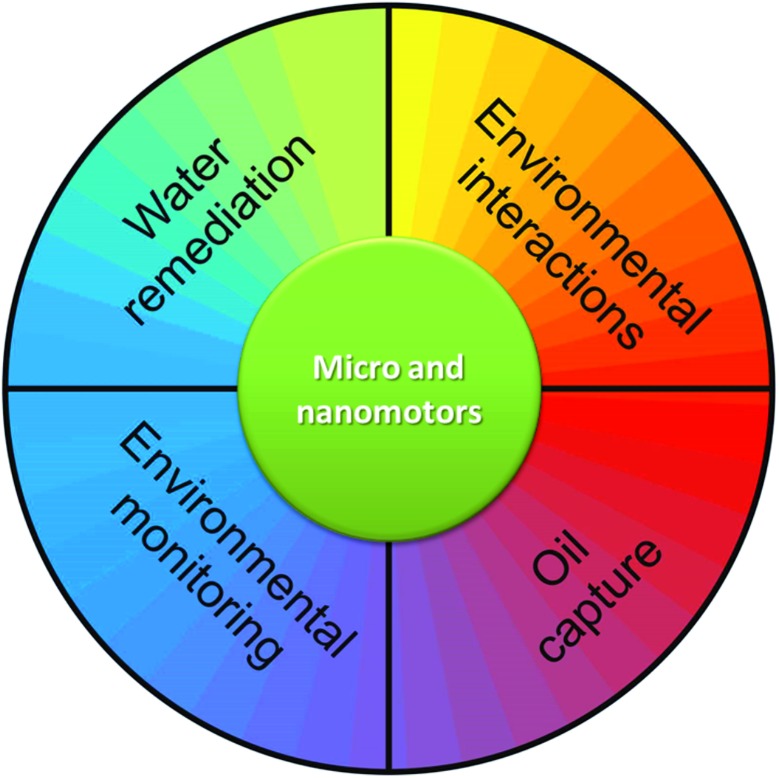
Environmental applications of micro- and nanomotors.

Although several groups are aiming at the use of catalytic nanomotors in the biomedical field, the current reduced biocompatibility of the fuel employed thus far for locomotion (H_2_O_2_ and/or hydrazine) still limits their realistic use. Enzymes decomposing other chemical fuels to generate either gradient of reactants and products around the nanomotors or gas generation may prove to be a good option that still needs to be further developed. Alternatively, two options are proposed: either to use bio-friendly powering methods, which must be compatible with biological fluids, *e.g.* magnetic or ultrasound propulsion, or to find applications for catalytic nanomotors in which the fuel employed does not limit their use. Among the latter option, environmental applications may be an important field to explore, where the use of hydrogen peroxide in particular is not controversial and is sometimes employed as co-reagent. In this paper, we summarize recent progress toward environmental applications of micro- and nanomotors and highlight crucial challenges for opening up a variety of new applications in the field (Fig. 1).

## 2. Environmental monitoring using nanomotors

**Fig. 2:**
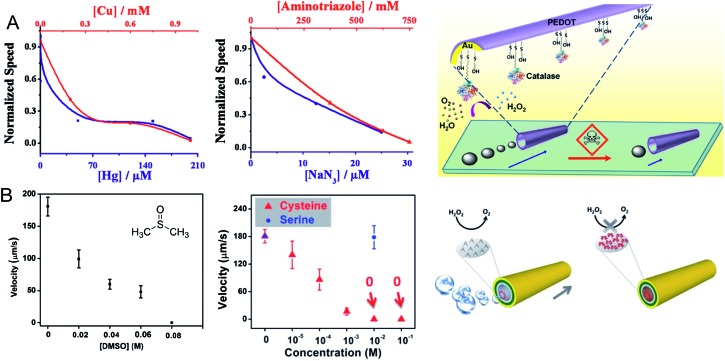
Nanomotors as active self-powered sensors. (A) Changes in the swimming behavior of the artificial micromotor (microfish) as a function of concentration upon 2 min exposure to Hg, Cu, sodium azide and aminotriazole. The solution contains 5% sodium cholate (NaCh), and 2% hydrogen peroxide (H_2_O_2_). Reprinted with permission from ref. 41. Copyright 2013, American Chemical Society. (B) Influence of the concentration of DMSO, cysteine and serine on the velocities of the microjets. Tracking data were obtained for a timescale of 10 seconds from 5 independently running experiments in order to get the average speed. The solution contains 1% sodium dodecyl sulfate (SDS) and 9% hydrogen peroxide (H_2_O_2_). Both schematics on the right panels illustrate the poisoning of micromotors – either enzymatic or Pt-based – upon presence of particular analytes. Reprinted with permission from ref. 44. Copyright 2013, Royal Society of Chemistry.

Earlier last year, nanomotors demonstrated the capability to detect and sense water quality where they swim. For instance, Orozco *et al.* described artificial enzymatic micromotors for water quality testing.^41^ The biocatalytic decomposition of the hydrogen peroxide fuel takes place at the inner enzymatic catalase layer of the microtube and generates oxygen bubbles, as previously reported by Sanchez *et al.* ^42^ In the presence of enzyme inhibitors (heavy metals, sodium azide) the bubble frequency decreases, and the change in motility of the artificial microfish is directly correlated to the concentration of the pollutant (Fig. 2A). A very recent publication from Moo *et al.* reported the specific response to Pb^2+^ in water, compared with Cd^2+^, by using enzyme-free Cu/Pt bimetallic microtubular motors.^43^ The authors take advantage of the different adsorption rates of those heavy metals on the catalytic Pt layer, *i.e.* Pb^2+^ is strongly adsorbed on platinum, whereas Cd^2+^ presents weaker interaction. Consequently, Pt-based microjets are more sensitive to poisoning with Pb^2+^ than with Cd^2+^, resulting in a different decrease in the speed of the micromotors.

Pumera and co-workers reported the use of micromotors to sense the presence of certain molecules in an aqueous environment.^44^ For instance, chemical quenchers of HO˙ radicals, such as dimethyl sulfoxide (DMSO), reduce the generation of O_2_ produced by microjet motors when H_2_O_2_ is decomposed in a disproportionation reaction *via* a radical pathway (Fig. 2B). Moreover, amino acids containing thiol groups such as cysteine and methionine, and peptides like glutathione can poison the Pt catalyst, which can have a significant effect on the motility of the bubble propelled micromotors, both rolled-up and electrochemically deposited microtubes (Fig. 2B, right plot). Electrolytes such as Na^+^, K^+^, Ca^2+^, Cl^–^, SO_4_^2–^ and phosphates, uric acid, and blood proteins such as bovine serum albumin (BSA), beta-globulin and glucose oxidase enzymes can be detected at small concentrations in the solution by monitoring the change in speed of the nanomotor that enables the monitoring of the concentration of these compounds.^45–47^ Although such speeds are dramatically reduced in blood samples,^48^ nanomotors can overcome viscosity and passivation effects when these samples are warmed to physiological temperatures.^49^

**Fig. 3:**
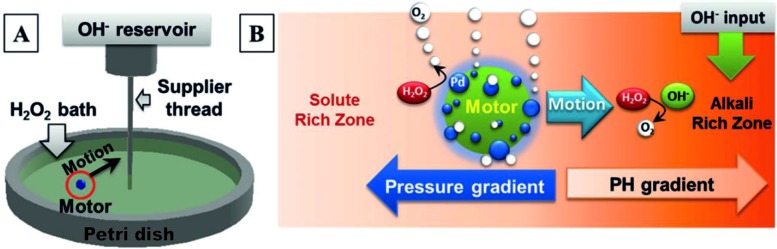
pH taxis of catalytic micromotors. (A) Schematic diagram of the experimental setup used. (B) Schematic description of the depletion mechanism. The solute H_2_O_2_ molecules migrate from the solute rich zone (left) towards the motor surface and react with Pd NPs to produce O_2_ bubbles. In the alkali rich zone (right), the solute concentration further decreases due to OH^–^ catalyzed volumetric reactions. Reprinted with permission from ref. 50. Copyright 2013, Royal Society of Chemistry.

Catalytic micro/nanomotors can also be used as pH sensors, taking advantage of their pH taxis capabilities. In a recent publication, Dey *et al.* reported a catalytic, self-propelled polymeric microsphere containing randomly distributed Pd nanoparticles (NPs) that showed pH taxis behaviour.^50^ Their experimental results revealed that the rate constant of the catalytic decomposition of H_2_O_2_ on the Pd catalytic sites increased with the pH of the medium, moving the micromotor with increasing speed from the lower pH regions in the aqueous solution towards that of the higher (Fig. 3).

## 3. Effect of the environment on the motion of nanomotors

**Fig. 4:**
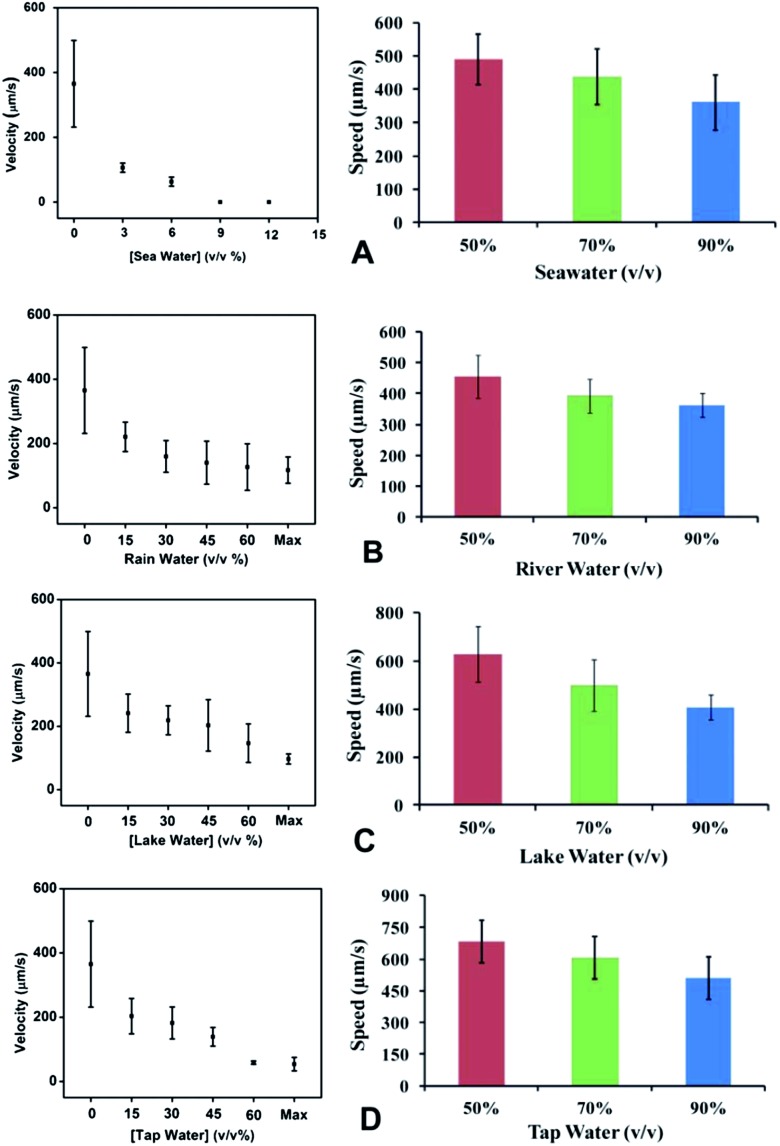
Motion of the catalytic microjets, prepared by the electrochemical template deposition method, in various types of waters, including (A) seawater, (B) rain and river water, (C) lake water and (D) tap water. Left column: Cu/Pt catalytic microjets, temperature of 23 °C, 3% (wt) H_2_O_2_ and 1 (wt)% SDS. Right column: (PEDOT)/Pt microtubes, 3% (w/v) H_2_O_2_, 3% (w/v) sodium dodecyl sulfate. Temperature is not stated. Reprinted with permission from ref. 51 and 52. Copyright 2013, Royal Society of Chemistry.

The surroundings in which micromotors swim are a crucial parameter for the motion of catalytic nano-micromotors. Viscosity, ions and analytes contained in the solutions in which nanomotors are swimming, can alter the dynamics of nanomotors, modifying their speed. Hence, the poisoning of the nanomotors is employed as an indirect measurement of the pollutant species by those self-propelled devices. In a recent work, Pumera and coworkers explored the influence of various types of water on the locomotion of catalytic tubular Cu/Pt micromotors, including tap water, rain water, lake water and sea water.^51^ The studies were carried out by fixing the concentrations of H_2_O_2_ and the surfactant, in addition to varying the concentration of the water sample, which was diluted with distilled water. In all cases, an increase of the concentration of real water samples caused a decrease in the bubble ejection and motility of the microjets (Fig. 4, left plots). Determining the ionic composition of the water samples, the authors concluded that the amount of inorganic ions in aqueous solution directly correlated to the decreased locomotion of micromotors. It is important to note that no motion was observed when the Cu/Pt catalytic microjets were immersed in seawater containing 3% H_2_O_2_ and 1% surfactant. Further experiments with an increasing concentration of sodium chloride (NaCl) in distilled water showed a similar decrease in the performance of microjets. Contrary to previous observations,^25^ the motion of catalytic bubble microjets was, in that case, highly influenced by the salt concentration in the environment. Despite the challenges found in the locomotion of Cu/Pt bimetallic microjets, Gao *et al.* reported propulsion of electrodeposited polymer/platinum microtubular motors, with a polymer-based outer layer composed of poly(3,4-ethylenedioxythiophene) (PEDOT) even in 90% concentrated water samples, including tap water, rain water, lake water and seawater, (Fig. 4, right plots).^52^ Although these micromotors self-propel in different real-life water sources, one should note that their speeds, compared with control experiments in deionized water (containing 3% hydrogen peroxide), are significantly reduced (980 μm s^–1^ in DI water *vs.* 489 μm s^–1^ in 50% v/v DI water–seawater). Thus, the different results presented by the two research groups are not totally contradictory and may arise from the difference in the material composition of the micromotors, and perhaps caused by the difference in their speeds in DI water containing H_2_O_2_. For example, the PEDOT/Pt bilayer micromotors have shown very high speeds (980 μm s^–1^ in 3% H_2_O_2_ in distilled water). This speed is about 3 times higher than the speed of bimetallic Cu/Pt micromotors in similar conditions (365 μm s^–1^ in 3% H_2_O_2_ and 1% sodium dodecyl sulfate as surfactant in distilled water).

## 4. Towards removal of oil from water

**Fig. 5:**
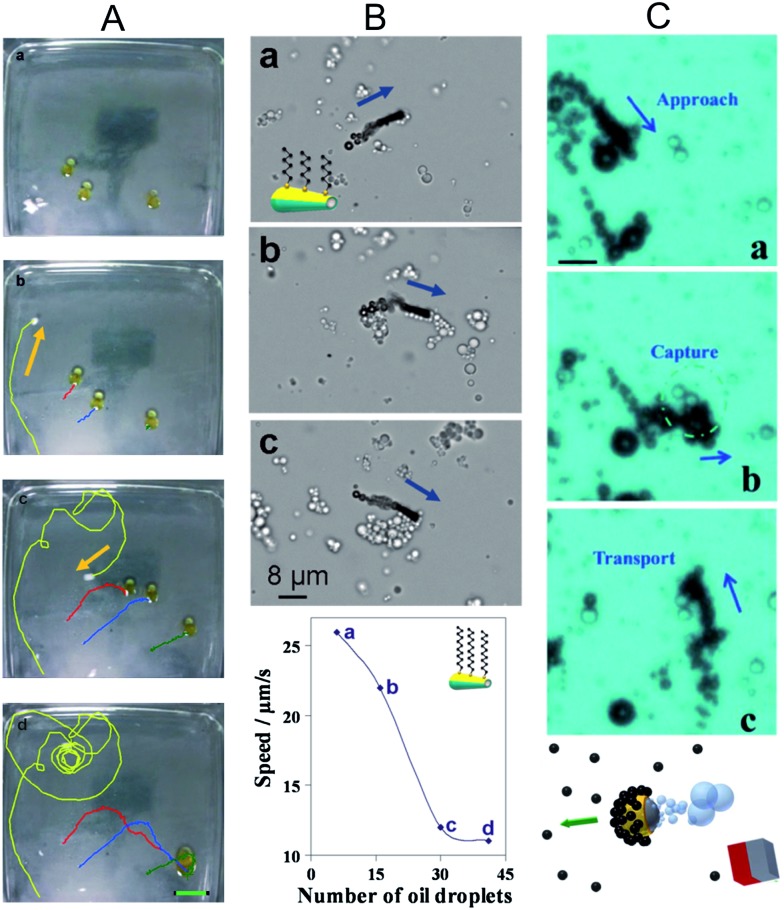
Oil capture and dispersion using micro- and nanomotors. (A) SDS/PSf capsule inducing movement onto oil droplets. Images taken at (a) *t* = 0, (b) 0.8, (c) 3.5 and (d) 7.0 s. The three oil droplets merge in 7.0 s. Scale bar 2.24 cm. Reprinted with permission from ref. 36. Copyright 2011 Wiley-VCH. (B) Catalytic microjets with hexanethiol-modified hydrophobic walls capturing oil droplets and transporting them. The plot shows the reduction of dodecanethiol-modified microjet speed with the number of oil droplets transported. Reprinted with permission from ref. 54. Copyright 2012 American Chemical Society. (C) Janus micromotor driven by Mg in water solutions. Similar to (B), the micromotors contain hydrophobic caps for oil capture. Reprinted with permission from ref. 55. Copyright 2013 American Chemical Society.

The surface modification of some types of nanomotors allows them to capture oil from contaminated waters. Research by Pumera and co-workers described a sodium dodecyl sulfate (SDS)-loaded polysulfone (PSf) capsule that was used to shepherd several oil droplets and to merge them, cleaning the surface of the water.^36^ The driving force of self-propulsion is based in the Marangoni effect. The PSf capsule was cast by dropping 5 μL of a solution of PSf in *N*,*N*-dimethylformamide (DMF) onto the surface of an aqueous solution, creating a millimeter-sized PSf porous capsule. The DMF in the capsule was released slowly and asymmetrically through its pores, leading to self-propulsion. When the SDS/PSf capsule was exposed to the water surface where several oil droplets were previously placed, the collective motion of the oil droplets induced by the SDS/PSf capsule was observed (Fig. 5A). Further improvements of the same group explored the incorporation of different surfactants to the PSf capsule, in order to induce motion of oil droplets.^53^ An increase in the concentration of surfactant in the capsule causes a rise in the velocity of the repulsed oil droplet, with SDS producing the fastest propulsion speed.

At smaller sizes, the first example of using functionalized catalytic micromotors for removing oil from water was reported by Wang's group.^54^ For this application, catalytic microtubular Au/Ni/PEDOT/Pt motors were fabricated by electrochemical deposition and further functionalized with alkanethiols to form a hydrophobic monolayer on the outer gold surface of the microtube. The strong interactions between the alkanethiol chains and oil from the solution, enable micromotors to capture oil droplets and transport them. The influence of the alkanethiol length was examined by functionalizing the micromotor with self-assembled monolayers (SAMs) of different alkanethiol lengths (C6, C12 and C18). The experimental observations illustrated a weak interaction of the micromotor with the oil droplet by using C6, but a stronger interaction was observed when C12 was used. However, further modification of micromotors with C18 hardly displayed motion due to the blocking of the inner catalytic Pt layer. As expected, the polarity of the head functional group influenced the interaction between the micromotor and the oil droplet. Another approach to capture oil from water, which was also based on SAM modification, was the use of self-propelled magnesium Janus particles in seawater samples.^55^ The magnesium particles were coated by nanometric layers of Ti, Ni and Au and further functionalized with octadecanethiol (Fig. 5C). The driving force of self-propulsion is based on the redox reaction of magnesium and water, which produces hydrogen bubbles and magnesium hydroxide as a byproduct. Although an oxide passivation layer can be rapidly formed on the Mg surface and, consequently, hamper the self-propulsion mechanism, Gao *et al.* found that the presence of the gold layer and chloride ions allow the hydrogen generation reaction to proceed. Note that Au plays a crucial role in the H_2_ generation due to the macro-galvanic corrosion mechanism, *i.e.* one metal corrodes preferentially to another when two metals are in contact. In this particular case, the formation of hydrogen bubbles will stop as soon as the Mg-based body of the motor is fully consumed. Since chloride-rich environments are required for proper performance of this motor, seawater appears to be a suitable media in which this particular type of micromotor can actuate. However, the lifespans of Mg-based micromotors appear to be a drawback for the development of practical applications for these microdevices.

## 5. Water remediation mediated by nanomotors

**Fig. 6:**
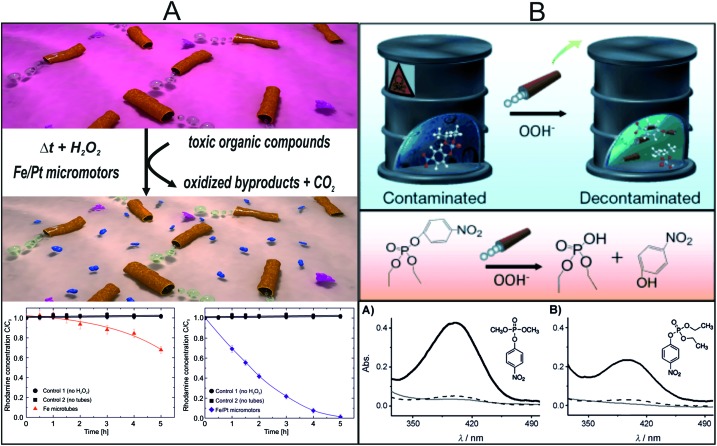
Micromotors employed for water remediation. (A) Removal of toxic organic compounds from aqueous solutions with Fe/Pt micromotors in H_2_O_2_ solutions. Pink molecules represent Rhodamine 6G (Rh6G); the small blue molecules represent oxidized compounds and carbon dioxide as reaction products. Plots show a quantitative comparison of Rh6G degradation by pure iron (Fe) tubes (red triangles) and by catalytically active Fe/Pt tubes (blue diamonds) over 5 h. Reprinted with permission from ref. 9. Copyright 2013, American Chemical Society. (B) Illustration of a micromotor-based accelerated oxidative detoxification of OP nerve agents. The micromotor-based strategy allows for rapid detoxification of chemical threats under mild conditions, and involves the *in situ* generation of OOH^–^ nucleophiles. Plots show the efficiency of the decontamination process by measuring the absorbance of the p-NP reaction product obtained in the presence (black line) and absence (grey line) of the micromotors, and with the motors not moving (dashed line). Reprinted with permission from ref. 10. Copyright 2013, Wiley-VCH.

Despite recent advances in environmental studies with micromotors that combine motion and water treatments, there continues to be a need for reusable and multifunctional micro- and nanomotors with extended lifespans that can degrade efficiently a variety of pollutants. In a recent work, Sanchez and co-workers illustrated for the first time the ability of self-propelled micromotors to oxidize organic pollutants in aqueous solutions.^9^ The novelty of the work lies in the synergy between the internal and external functionality of the micromotors. In particular, these multifunctional microtubular motors use hydrogen peroxide as a fuel for locomotion, produced by the generation of O_2_ bubbles in the internal Pt layer, while actively degrading organic pollutants in solution due to the useful function of the external Fe wall as active material that enables water remediation (Fig. 6A). The mechanism of degradation is based on Fenton's reaction relying on spontaneous acidic corrosion of the iron metal surface of the micromotors in the presence of H_2_O_2_, which acts both as a reagent for the Fenton reaction and as a fuel to propel the micromotors. These findings have proven that the advantage of the newly designed micromotors based on their capacity to provide, owing to their motion, enhanced catalytic reactions. The importance of these findings opens the way to fabricate autonomous microscopic cleaning systems that can work without external energy input and in a much faster way than their static counterparts. The micromotors offer this ability to move the catalyst (ions) around without external actuation or additional catalysts (iron salts) to achieve water remediation, removal of organic dyes, *etc.* Remarkably, oxidation is achieved even in the absence of a surfactant due to the double functionality of the Fe/Pt microtubular motors. Micromotors can still self-propel without surfactants, although at slower speeds. Yet, their active motion boosts the degradation of model pollutants such as Rhodamine 6G.

Almost at the same time, parallel efforts by Wang *et al.* reported the use of PEDOT/Pt micromotors to aid in the oxidation chemical threats.^10^ In that work, the oxidative decontamination of the organophospate (OP) nerve agent in the presence of hydrogen peroxide was accelerated by the presence of the self-propelled micromotors that contributed to an efficient fluid mixing without the need of external mechanical stirring (Fig. 6B). Similar to the work described above, hydrogen peroxide has double functionality, acting as a fuel to propel the micromotors and as an oxidizing reagent for decontaminating the aqueous solution. The authors also studied the ability of employing these micromotors to accelerate the decontamination of several OP pesticides with analogous molecular structures, including methyl paraxon (MP), ethyl paraxon (EP) and bis(4-nitrophenyl)phosphate (b-NPP). In all cases, oxidation was not observed when the same mixture with OP pollutants and H_2_O_2_ reacted for the same duration without the presence of the micromotors in the aqueous solution. Interestingly, no oxidation signals were detected when surfactant was not added in the solution, rather in the presence of micromotors. The authors observed that increasing the number of micromotors immersed in the treated solution, a decontamination of 100% can be achieved by using significantly shorter reaction times and lower peroxide concentrations, compared to common neutralization processes of chemical warfare agents that require mechanical agitation.

## 6. Conclusions and future challenges

The recent progress on artificial nanomotors is opening up initial proof-of-concept environmental applications. This review has highlighted prospects and challenges in transferring recent advances in micro and nanomotors toward practical environmental applications. While significant advances have been made during the past decade towards demonstrating initial proof-of-concept environmental studies, the achievement of practical environmental applications requires further innovation. Current technologies are inadequate for meeting the demand in scaling up the processes reported in the remediation of large amounts of polluted water, and much more work is required. Several issues need to be solved before scaling one step toward practical application. For instance, the lifespan of the multifunctional micromotors is limited to the remaining materials in its body that were involved/consumed in oxidation or locomotion reactions (Fe, Mg). Other drawbacks could be the poisoning of the platinum layer due to the presence of compounds in the wastewater that can bond chemically to the active surface sites of the catalyst, or a high viscosity of treated wastewater that could hamper the motion of the micromotors.

An expected increase in innovation in this research field and a predictable further development of new capabilities introduced in self-propelled micro- and nanomotors will provide a myriad of environmental possibilities to perform more complex and demanding operations. We envision future nanomotors to deal especially at the microscale and difficult-to-reach environments. As other types of nanomotors such as DNA nanomachines can already detect pH changes inside living cells,^56^ future research could be envisioned through the integration of those DNA sensors with artificial self-propelled nanomotors. These foreseeable new micro- and nanomotors would eventually revolutionize environmental monitoring and water remediation technologies, in an effort to improve the quality of life.
